# Vascular contraction of umbilical arteries of pregnant women with preeclampsia

**DOI:** 10.61622/rbgo/2024AO02

**Published:** 2024-03-15

**Authors:** Gabriela Morelli Zampieri, Priscila Rezeck Nunes, Joelcio Francisco Abbade, Carlos Alan Dias, Valeria Cristina Sandrim

**Affiliations:** 1 Universidade Estadual Paulista "Julio de Mesquita Filho" Botucatu SP Brazil Universidade Estadual Paulista "Julio de Mesquita Filho", Botucatu, SP, Brazil.

**Keywords:** Preeclampsia, Glibenclamide, Vascular contractility, Umbilical arteries

## Abstract

**Objective:**

Potassium channels have an important role in the vascular adaptation during pregnancy and a reduction in the expression of adenosine triphosphate-sensitive potassium channels (Katp) has been linked to preeclampsia. Activation of Katp induces vasodilation; however, no previous study has been conducted to evaluate the effects of the inhibition of these channels in the contractility of preeclamptic arteries. Glibenclamide is an oral antihyperglycemic agent that inhibits Katp and has been widely used in vascular studies.

**Methods:**

To investigate the effects of the inhibition of K_atp_, umbilical arteries of preeclamptic women and women with healthy pregnancies were assessed by vascular contractility experiments, in the presence or absence of glibenclamide. The umbilical arteries were challenged with cumulative concentrations of potassium chloride (KCl) and serotonin.

**Results:**

There were no differences between the groups concerning the maternal age and gestational age of the patients. The percentage of smokers, caucasians and primiparae per group was also similar. On the other hand, blood pressure parameters were elevated in the preeclamptic group. In addition, the preeclamptic group presented a significantly higher body mass index. The newborns of both groups presented similar APGAR scores and weights.

**Conclusion:**

In the presence of glibenclamide, there was an increase in the KCl-induced contractions only in vessels from the PE group, showing a possible involvement of these channels in the disorder.

## Introduction

Preeclampsia (PE) is a hypertensive disorder of pregnancy that complicates 2 – 8% of pregnancies worldwide.^(1)^ It is one of the leading causes of maternal and neonatal morbidity and mortality^(2,3)^ and can result in higher risks of future cardiovascular diseases.^(4)^ The etiology of the disease is still unknown, but it can be characterized by an abnormal vascular response to placentation associated with increased systemic vascular resistance, platelet aggregation, coagulation system activation and endothelial dysfunction.^(5)^

Studies have shown vasoreactivity differences in fetal vessels in pregnancies complicated by preeclampsia, however there is a lot of discrepancy in the results, with some articles showing increased vascular responses in PE and others showing a decrease in the responses to the same vasoactivators.^(6–10)^ Since placental vascular dysfunction plays an important role in the physiopathology of PE,^(11)^ the different responses to vasoactive substances in the fetal vessels of these patients could bring insight to the development of the disease.

Potassium channels have an important vasodilatory role in the vascular adaptation during pregnancy, promoting a balance between pro-contractile and pro-dilatory responses.^(12)^ Activation of adenosine triphosphate-sensitive potassium channels (K_atp_) inhibits the release and refilling of Ca^2+^ from intracellular stores, one of the primary factors for smooth muscle contraction.^(13)^ These channels are regulators of vascular responses during pregnancy, having their actions enhanced by several vasodilators and decreased by vasoconstrictors such as angiotensin II, serotonin and endothelin-1.^(12)^ In fact, the deletion of the subunit Kir6.1 of K_atp_ channels resulted in hypertension in mice^(14)^ and recent articles have shown a decrease in the expression of these channels in umbilical arteries of pregnant women who had hypertension during pregnancy and gestational diabetes.^(15,16)^ Furthermore, the expression of the subunit SUR2B of these channels is reduced in umbilical arterial smooth muscle cells of women with severe PE.^(17)^

K_atp_ channels also play an important role in the cellular alterations that occur during hypoxia and ischaemia,^(18)^ pathophysiological states that are present in the placenta during PE, and the impairment of these channels has been theorized to be a factor in the pathogenesis of PE.^(13)^ However, to our knowledge no previous study has evaluated the inhibition of K_atp_ channels in vessels of women with PE. Glibenclamide (glyburide) is an oral antihyperglycemic agent of the second generation of sulfonylureas that increases insulin secretion by pancreatic β cells by inhibiting K_atp_ channels^(19)^ and it has been widely used as a K_atp_ channel inhibitor in many vascular studies.^(20–24)^

The clear role of K_atp_ channels in the cellular mechanisms of vasodilation and the differences found in the expression of these channels in hypertension during pregnancy, gestational diabetes and PE suggests that K_atp_ channels might be involved in the abnormal vascular responses we see in these disorders. Thus, the use of a K_atp_ channel inhibitor in vascular contractility experiments with umbilical arteries of preeclamptic patients may help investigate the role of these channels in the patterns of vasoconstriction in PE.

Therefore, the aim of the current study was to investigate if the fetal vessels in PE exhibit excessive vascular responses to serotonin and potassium chloride and the effects of the inhibition of K_atp_ on these responses. For this, umbilical arteries of preeclamptic women and women with healthy pregnancies were assessed by vascular contractility experiments, in the presence or absence of glibenclamide.

## Methods

All studied patients had deliveries in Maternity Hospital of the *Hospital das Clínicas* of the Faculty of Medicine of Botucatu. Eleven patients were included in the study, six were diagnosed with PE and five had healthy pregnancies, from December 2021 to October 2022. Gestational age was determined by the date of the last menstrual period and confirmed by early ultrasound examination. Patients that presented any obstetric or clinical intercurrences, like the use of drugs or alcohol, twin pregnancy, fetal malformation, renal or infectious diseases, were excluded from the study.

The following compounds were used in this study: 5-hydroxytryptamine (H-7752, Sigma-Aldrich, Saint Louis, MO, USA), potassium chloride (1526-1, Dinamica chemicals, São Paulo, Brazil) and glibenclamide (G0639, Sigma-Aldrich, Saint Louis, MO, USA) Glibenclamide was dissolved in dimethyl sulfoxide (13-0091-01, LGC Biotecnologia, São Paulo, Brazil) and then diluted in deionized water.

Approximately 15 to 30 minutes after delivery, fragments of 10 cm were removed from the umbilical cord. The fragments were transported in a becker containing cold Krebs-Henseleit solution (composed of: NaCl 119.0 mM; KCl 4.7 mM; CaCl_2_ 2.5 mM; KH_2_PO_4_ 1.2 mM; MgSO_4_ 1.2 mM; NaHCO_3_ 25 mM; Glucose 11mM). Adipose and connective tissue were surgically removed from the umbilical artery and the artery was then cut into rings of approximately 3-4mm. The artery rings were placed in a 10 ml organ chamber containing Krebs-Henseleit solution, gassed continuously with a mixture of 95% O_2_ and 5% CO_2_ and maintained at 37ºC. The artery rings were suspended in parallel by two wire hooks that cross the lumen of the vessel, one hook was fixed to a stationary support and the other was connected to an isometric force transducer.

Before starting the vascular reactivity experiment, the rings were allowed to equilibrate for 120 minutes with optimal basal tension of 2g. The solution in the organ chambers was changed every 15 minutes during the entirety of the experiment. Changes in vascular tone were recorded using FORT10 isometric force transducers connected to a PC-based MP100 System and analyzed off-line using AcqKnowledge version 3.5.7 software (Biopac Systems Inc., Goleta, CA).

After tissue equilibration, each umbilical artery ring was challenged twice by 96 mM of potassium chloride (KCl); the rings that showed no response to KCl were discarded. With the confirmation of vessel viability, the rings were then challenged with cumulative concentrations of KCl (10-120 mM) and serotonin (5HT) (10^−10^ a 10^−4^ mol/L). The vessels were incubated with glibenclamide for 15 minutes and the same vascular challenges were performed in the presence of the drug.

For the vascular reactivity experiments, individual concentration-contraction curves were constructed; sigmoidal curves were fitted to the data using the least square method, and the values of the negative logarithm of the concentration that evoked 50% of the maximal response (pEC_50_) were calculated, and comparisons among maximum responses (E_max_) and pEC_50_ values were analyzed. The value of p < 0.05 was considered significant. To compare E_max_ and pEC_50_ values, we used the Student *t*-test. Student *t*-test was also used to compare the average contraction of the studied groups and two-way ANOVA was used to analyze the responses point-by-point.

All pregnant women signed the Free and Informed Consent Form, and the study was approved by the Research Ethics Committee of the Botucatu School of Medicine (nº 4961945, approved on 9 September 2021) (CAAE: 50963021.0.0000.5411).

## Results

### Clinical parameters of the studied patients

The clinical parameters of the patients whose umbilical cords were collected for vascular reactivity experiments are displayed in table 1.

**Table 1:** Clinical parameters

Parameters	Healthy pregnancies	PE	p-value
n	5	6	–
Age	26.0 ± 1.6	27.5 ± 1.7	0.4285
Caucasian (%)	100.0	83.3	0.3384
Primiparae (%)	60.0	50.0	0.7401
Smoker (%)	0.0	16.7	0.3384
GAD (weeks)	39.0 ± 0.5	37.3 ± 0.6	0.0786
SBP (mm Hg)	122.0 ± 3.7	144.6 ± 7.5	0.0323
DPB (mm Hg)	80.0 ± 3.0	93.0 ± 3.2	0.0173
BMI (kg/m2)	30.0 ± 1.9	36.2 ± 1.4	0.0266
Uric acid (mg/dl)	N/D	6.1 ± 1.1	–
Hemoglobin (g/dl)	N/D	12.6 ± 1.1	–
Hematocrit (%)	N/D	38.1 ± 3.1	–
Platelets (x10^3^ /mm^3^)	N/D	218.2 ± 50.7	–
Creatinine (mg/dl)	N/D	0.5 ± 0.2	–
Newborn weight (g)	3190.0 ± 60.6	2875.0 ± 253.4	0.4285
APGAR Score 1 (1 min) < 7 (%)	16.6	0.0	0.2963
APGAR Score 2 (5 min) < 7 (%)	0.0	0.0	>0.9999

GAD - gestational age at delivery; SBP - systolic blood pressure; DBP - diastolic blood pressure; BMI - body mass index; ND - not determined. Values are mean ± standard error and frequency (percentage) for categorical variables. Parametric variables were compared by Student t-test and non-parametric by Mann-Whitney test. Categorical variables were compared by contingency table

There were no differences between the groups concerning the maternal age and gestational age of the patients. The percentage of smokers, caucasians and primiparae per group was also similar. On the other hand, blood pressure parameters were elevated in the preeclamptic group. In addition, the preeclamptic group presented a significantly higher body mass index. The newborns of both groups presented similar APGAR scores and weights. Three of the patients studied presented superimposed PE (the development of PE in patients with chronic hypertension before pregnancy) and only one of the patients had sings of severe preeclampsia before delivery.

### Differences in the KCl-induced contraction and serotonin-induced contractions between healthy pregnancies and PE

**Figure 1:**
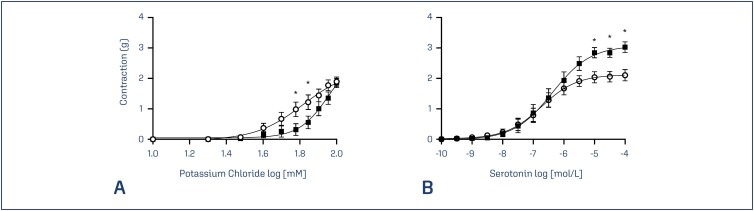
Concentration-response curves to KCl (A) and to serotonin (B) in rings of umbilical arteries from healthy pregnancies (*open circles*) and from pregnancies complicated by PE (*closed squares*). Symbols represent significant differences between the groups in the contractile response to 60 mM (p = 0.0375) and 70 mM (p = 0.0401) of KCl and significant differences between the groups in the contractile response to 10^−5^ mol/L (p = 0.0185), 10^−4.5^ mol/L (p = 0.0239) and 10^−4^ mol/L (p = 0.0028) of serotonin. Data presented as mean ± SEM

The concentration-response curves to potassium chloride (10-120 mM) of umbilical arteries of healthy pregnant patients and preeclamptic patients were constructed and compared (Figure 1). The average contraction value of the arteries derived from the preeclamptic patients was diminished (p = 0.0479), showing a significant reduction in the responses to the concentrations of 60 mM and 70 mM of KCl (Figure 1A). The pEC_50_ values for the group with PE were higher in comparison to the healthy group, yet the maximum responses were similar between the groups (Table 2). The average contraction value of the preeclamptic arteries was augmented when cumulative concentrations of serotonin (10^−10^-10^−4^ mol/L) were used (p = 0.0331), showing a significant increase in the responses to the concentrations of 10^−5^, 10^−4,5^, and 10^−4^ mol/L (Figure 1B). The pEC50 values were similar between the groups and the maximum responses to serotonin-induced contraction were higher in the preeclamptic group (Table 2).

**Table 2:** Negative logarithm of the concentration that evoked 50% of the maximal response (pEC_50_) and maximal responses (E_max_) were recorded for KCl and serotonin-induced contractions in the absence and presence of glibenclamide for umbilical arteries from pregnancies complicated by PE and from healthy pregnancies

PEC_50_ AND E_MAX_	Healthy pregnancies	PE	PE	
			GB 3.6 µg/mL	GB 5.0 µg/mL
KCl E_max_	2.09 ± 0.12	2.23 ± 0.12	3.28 ± 0.24 #	3.22 ± 0.18 &
KCl pEC_50_	1.75 ± 0.03	1.92 ± 0.01*	1.76 ± 0.10#	1.79 ± 0.06 &
5HT E_max_	2.12 ± 0.18	3.15 ± 0.18*	3.03 ± 0.41	3.11 ± 0.32
5HT pEC_50_	6.82 ± 0.23	6.43 ± 0.19	6.17 ± 0.42	5.95 ± 0.47

Data is expressed as mean ± SEM. Symbols represent significant differences between healthy pregnancies and pregnancies complicated by PE

* (p < 0.005, Student t-test) or differences between pregnancies complicated by PE with and without glibenclamide (*#,&* p < 0.005, Student t-test)

### Effects of glibenclamide in the vascular responses of umbilical arteries of preeclamptic patients

**Figure 2:**
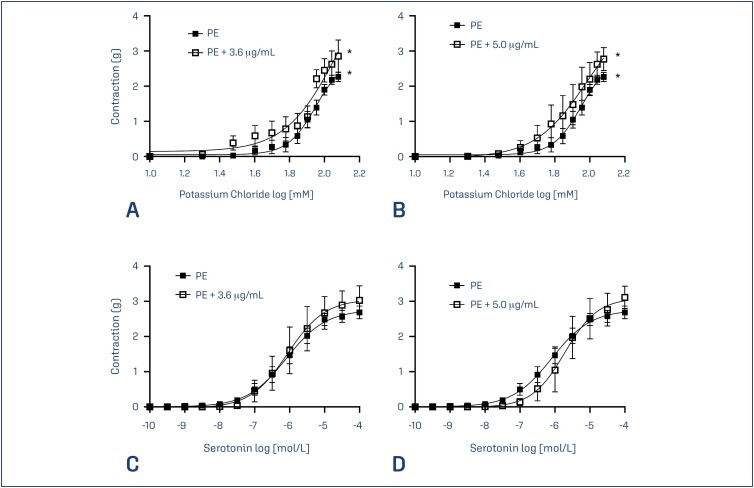
Concentration-response curves to KCl in rings of umbilical arteries from pregnancies complicated by PE without glibenclamide (*closed squares*) and from pregnancies complicated by PE incubated with glibenclamide (*open squares*) (A, B). Concentration-response curves to serotonin in rings of umbilical arteries from pregnancies complicated by PE without glibenclamide (*closed squares*) and from pregnancies complicated by PE incubated with glibenclamide (*open squares*) (C, D). Symbols represent significant differences in the contractive response between the groups. Data presented as mean ± SEM

The concentration-response curves to KCl (10-120 mM) of umbilical arteries of preeclamptic patients in the presence of glibenclamide were constructed and compared to the curves obtained without the drug (Figure 2). Student *t*-test was applied and increases in the average contraction of the arteries were observed after the incubation with 3.6 and 5.0 µg/mL of glibenclamide (p = 0.0002 and p = 0.0006, respectively). The maximum response to KCl was augmented and the pEC_50_ values diminished by the addition of glibenclamide (Table 2). The concentration-response curves to serotonin (10^−10^-10^−4^ mol/L) of umbilical arteries of preeclamptic patients in the presence of glibenclamide were also constructed and compared to the curves obtained without the drug. Glibenclamide showed no effects on the vascular response to serotonin.

### Effects of glibenclamide in the vascular response of umbilical arteries of healthy pregnant patients

Glibenclamide showed no effects on the vascular response to potassium chloride or to serotonin in the healthy umbilical arteries.

## Discussion

In the present study, the contractile responses to serotonin and KCl on human umbilical arteries from patients with PE and healthy pregnancies were examined. We also investigated the effects of glibenclamide, a K_atp_ channels inhibitor, on the serotonin and KCl-induced contractions of both groups.

Umbilical cord vessels have no innervation, thus blood flow control depends on existing vasoactive substances in the circulation, such as serotonin and ions such as K^+^ and Ca^2+^.^(25,26)^ Serotonin is one of the most important vasoconstricting agents of the umbilical artery, being responsible for the physiological closure of the artery after birth.^(26)^ Studies have already demonstrated elevated levels of serotonin in placentas^(27–29)^ urine^(30,31)^ and in the plasma^(32,33)^ from patients with PE when compared with controls. There were also studies that demonstrated a relationship between the elevation of serotonin levels in the plasma of pregnant women and the severity of PE^(34)^ and a higher expression of serotonin-7 receptors (5-HT_7_) in placentas of preeclamptic patients.^(35,36)^

Given the important role of serotonin in pregnancy and the observed changes in its levels in PE, it is possible that there are changes in the response to serotonin in umbilical vessels of pregnant women diagnosed with this syndrome. In our study, an increase in contraction was found in the umbilical arteries of pregnant women diagnosed with PE after stimulation with serotonin. Similar results that showed an increase in serotonin sensitivity in pregnant women with hypertension induced by pregnancy^(37)^ and in pregnant women with PE^(8)^ were found. However, there is conflicting data in the literature, other studies found no difference in the response to serotonin between healthy pregnant women and pregnant women with PE,^(38,39)^ and one study showed an increased response in the group of healthy pregnant women.^(7)^ It is worth mentioning that the studies that presented conflicting results used different methodological characteristics, such as smaller initial tension values and stabilization time and longer sample harvest times. In the standardization process performed in our laboratory before the beginning of the experiments, it was possible to observe a lower response to serotonin and KCl from the umbilical arteries when the initial tension was below 2g and when stabilization time was less than 2 hours, with some rings contracting less than 0.5g. [Data not shown].

The potassium chloride-induced contraction in the umbilical arteries is promoted by the influx of extracellular Ca^2+^ via voltage-gated Ca^2+^ channels and release of intracellular stores of Ca^2+^.^(13)^ In the reactivity assay, a decreased sensitivity to KCl was observed in the group with PE, which is consistent with results found in studies performed with an experimental model of PE in rats^(40)^ and also in a study with pregnant women that showed a higher threshold dose of KCl in patients with PE when compared to the control group.^(6)^

No difference was seen between the studied groups in the maximum response to KCl, which is in agreement with previous results found in the literature.^(6)^ The difference in sensitivity to KCl, demonstrating a delayed contraction compared to the control group, may possibly be explained by the impaired output of Ca^2+^ from its intracellular reserves. A study conducted by Haché et al demonstrated a reduced expression of the messenger RNA of genes implicated in the release of calcium into the cytosol such as inositol-1,4,5-triphosphate receptor (IP3R 1,2) and Ryanodine receptor (RyR 1,2,3) in placentas of patients with PE.^(41)^ An increased level of messenger RNA for sarcoendoplasmic reticulum Ca^2+^ ATPases (SERCA 1,2,3), which are responsible for calcium uptake in the cytosol,^(41)^ was also found. These alterations may be impairing the exit of Ca^2+^ to the cytosol, which would lead to a slower contraction in response to KCl.

The presented data shows how fetal vessels from patients with PE present distinct patterns of vascular responses. This offers important insights to the study of the disease, showcasing that it is not only the maternal vascular system that plays a role in PE, but the fetal vessels as well, for their vasoreactivity greatly influences the maternal-fetal blood flow.

Currently, there is still no consensus on the participation of ATP dependent potassium channels (K_atp_) on the contraction or relaxation of the human umbilical artery.^(42)^ Although, some recent articles found a decrease in the expression of these channels in umbilical arteries of pregnant women who had hypertension during pregnancy and gestational diabetes,^(15,16)^ and the expression of the subunit SUR2B of these channels is reduced in umbilical arterial smooth muscle cells of women with severe PE.^(17)^ which is an indication of their possible involvement in syndromes related to pregnancy. They also play an important role in various pathophysiological conditions including hypertension, diabetes, ischemia and hypoxia.^(43)^ The opening of potassium channels causes hyperpolarization of smooth muscle cells, and with this the closure of Ca^2+^ channels and reduction of cytosolic Ca^2+^, leading to relaxation and vasodilation.^(44)^ More specifically, in the human umbilical artery, vasodilation induced by cyclic monophosphate of guanosine is mediated by the activation of potassium channels.^(45)^

Glibenclamide is an antidiabetic agent from the sulfonylurea group whose main mechanism of action is the blockage of K_atp_ channels. The effect of glibenclamide on umbilical arteries of healthy pregnant women, of women with hypertension during pregnancy and of women with gestational diabetes are being studied,^(46–48)^ but its action on vessels of pregnant women with PE still needs to be elucidated. In healthy vessels, glibenclamide showed no effect on contractions induced by serotonin, bradykinin, histamine, and KCl,^(46–48)^ which corroborates with the findings of our study.

Furthermore, we observed that glibenclamide had no effect on the serotonin-induced contraction in the PE group. In umbilical arteries of pregnant women with pregnancy-induced hypertension and gestational diabetes, glibenclamide also promoted no changes to contractions induced by bradykinin^(47)^ or serotonin.^(48)^ However, in calcium free medium, the use of K_atp_ openers abolished serotonin-induced contractions in human umbilical arteries, showing that these channels do participate in the maintenance of vascular balance.^(13)^

In our results, the umbilical arteries of the group of patients with PE showed an increase in mean contraction and maximum response to KCl in the presence of glibenclamide, as well as a decrease in pEC_50_. This can be explained by the blockage of K_atp_ channels, which would lead to the opening of voltage-gated Ca^2+^ channels and increase in Ca^2+^ influx, adding to the increase in intracellular calcium already caused by the administration of KCl. It is important to note that this summation effect was not observed in healthy vessels, which may be a consequence of the higher expression of K_atp_ channels already mentioned. With a greater number of channels it is likely that the dose of glibenclamide used was not sufficient to cause an efficient blockage and so there was no effect on the healthy vessels. Also, it is already known that there is a lack of vasorelaxation mechanisms in vessels affected by PE^(5,11)^ which may be facilitating this sum of vasoconstricting actions.

It is important to mention that three of the studied patients had superimposed PE and that we did not have access to the history of use of antihypertensive drugs for any of the patients. This may have influenced the vascular reactivity studies, but the clinical parameters showed no evidence of large deviations between patients in the same group.

## Conclusion

In conclusion, the vessels of patients with PE showed a lower sensitivity to potassium chloride and a greater contraction to cumulative concentrations of serotonin. The umbilical arteries of patients with PE demonstrated increased KCl-induced contractions when incubated with glibenclamide. This study shows a need for further investigation of the involvement of K_atp_ channels in PE, as the inhibition of these channels had different effects on the preeclamptic vessels, showing that these channels might be implicated in the abnormal vascular responses found in this disorder and could be a therapeutic target for future research.
